# Preparation and Properties of 3D Printed Alginate–Chitosan Polyion Complex Hydrogels for Tissue Engineering

**DOI:** 10.3390/polym10060664

**Published:** 2018-06-14

**Authors:** Qiongqiong Liu, Qingtao Li, Sheng Xu, Qiujian Zheng, Xiaodong Cao

**Affiliations:** 1 School of Medicine, South China University of Technology, 382 Outer Ring Rd, Guangzhou 510006, China; 13242845287@163.com (Q.Liu); qingtaoli007@163.com or mcqtli@scut.edu.cn (Q.Li); 2 National Engineering Research Center for Tissue Restoration and Reconstruction, South China University of Technology, 382 Outer Ring Rd, Guangzhou 510006, China; 13662466914@163.com; 3 School of Material Science and Engineering, South China University of Technology, 381 Wushan Rd, Guangzhou 510641, China; 4 Guangdong General Hospital, 106 Zhongshan Second Rd, Guangzhou 510080, China

**Keywords:** 3D printed hydrogels, polyion complex, alginate, chitosan

## Abstract

Three-dimensional (3D) printing holds great potential for preparing sophisticated scaffolds for tissue engineering. As a result of the shear thinning properties of an alginate solution, it is often used as 3D printing ink. However, it is difficult to prepare scaffolds with complexity structure and high fidelity, because the alginate solution has a low viscosity and alginate hydrogels prepared with Ca^2+^ crosslinking are mechanically weak. In this work, chitosan powders were dispersed and swelled in an alginate solution, which could effectively improve the viscosity of an alginate solution by 1.5–4 times. With the increase of chitosan content, the shape fidelity of the 3D printed alginate–chitosan polyion complex (AlCh PIC) hydrogels were improved. Scanning electron microscope (SEM) photographs showed that the lateral pore structure of 3D printed hydrogels was becoming more obvious. As a result of the increased reaction ion pairs in comparison to the alginate hydrogels that were prepared with Ca^2+^ crosslinking, AlCh PIC hydrogels were mechanically strong, and the compression stress of hydrogels at a 90% strain could achieve 1.4 MPa without breaking. In addition, human adipose derived stem cells (hASCs) adhered to the 3D printed AlCh PIC hydrogels and proliferated with time, which indicated that the obtained hydrogels were biocompatible and could potentially be used as scaffolds for tissue engineering.

## 1. Introduction

Three-dimensional (3D) printing and bioprinting is a rapidly growing field that aims to develop sophisticated constructs for tissue regeneration. These approaches hold the potential to achieve functional tissue constructs by repairing the complex architecture and organization of native tissues. As such, the preparation complex structure with a similar native tissue is important [1]. Hydrogels are important materials for the preparation of 3D printed tissue-engineered scaffolds [2]. The precursor solution act as 3D printing ink for preparing hydrogels. Its viscosity and transitions process of sol to gel determines the shape fidelity and structure of 3D printed hydrogels [3]. Adding nanomaterial into 3D printing ink or preparing hydrogels with double networks are the regular methods [4,5,6], but the biocompatibility and degradation properties of 3D printed hydrogels that are used as scaffolds for tissue engineering cannot be guaranteed [7]. For the preparation of 3D printed, hydrogels act as scaffolds for tissue engineering, with a complex structure and high shape fidelity, however there are still many problems that need to be solved.

Sodium alginates, which are extracted from brown seaweed, are biocompatible polyanionic [8]. As a result of the shear thinning properties of the alginate solution, it is often used as precursor solution for 3D printed tissue-engineered constructs [9,10]. When an alginate solution was used for preparing tissue-engineered scaffolds, the structure and shape fidelity of the 3D printed hydrogels is usually difficult to guarantee, because the viscosity of the maximum concentration of alginate solution is still insufficient, the deposited filaments are easily fused and collapsed. In addition, the alginate hydrogels that are crosslinked with Ca^2+^ are mechanically weak [4], and the deposited filaments are easily collapsed because of gravity, which also has influence on the structure and shape fidelity of 3D printed hydrogels.

Acting as a biocompatible polyanionic, the alginate can also be crosslinked with polycation to obtain polyion complex (PIC). The mechanical strength of PIC hydrogels, prepared with two oppositely charged polyelectrolytes, is controllable through changing reactive ion pairs [11]. The mixing of bulk solutions of polycation and polyanion usually leads to inhomogeneous precipitation, where a strong PIC is formed at the interface of the two solutions, which quenches the further reaction [11,12]. As a result of the limited reactive ion pairs that are involved in the reaction, the crosslinking density of the biomaterial internal network is low. To solve this problem, Luo et al. [13] polymerized one of the polyelectrolytes from its monomers solution in the presence of another oppositely charged polymer at 1:1 charge ratio. The cationic monomer 3-(methacryloylamino) propyl-trimethylammonium chloride (MPTC) was homopolymerized in the first step and was then mixed with the anionic monomer sodium p-styrenesulfonate (NaSS). After the well dispersion, the anionic monomer is polymerized in the second step, to form soft PIC hydrogel. Tensile and compression tests indicate that the hydrogels that are formed by the oppositely charged polyelectrolytes are tough, self-healing, and rebuildable.

Chitosan is the only natural polycation polysaccharide, which is swelling, but it is insoluble in an aqueous solution. When the pH value of the aqueous solution is less than 4, chitosan is soluble and can react with anion or polyanion, such as alginate, to prepare the PIC hydrogels [14]. In this work, chitosan powders were added into the alginate solution as the 3D printing ink, and chitosan is swelling but insoluble in the alginate solution, which can effectively improve the viscosity of the alginate solution. After being treated with a hydrochloric acid (HCl) solution, the chitosan powders that were dispersed in am alginate solution were soluble and reacted with alginate, thus, 3D printed of AlCh PIC hydrogels were obtained. A molecule of HCl is a small molecule that diffuses easily, thus, the further reaction between two oppositely charged polyelectrolytes will not be quenched. With the increase of the reactive ion pairs, the mechanical strength of the 3D printed AlCh PIC hydrogel can be improved, and the 3D printed hydrogels with complex structures could also be prepared correspondingly.

## 2. Materials and Methods

### 2.1. Materials

Sodium alginate (Al, viscosity: 180–220 mp·s) and chitosan (Ch, viscosity: 100–200 mp·s, and the degree of deacetylation >95%) were both obtained from Aladdin. HCl was obtained from Huachengda Chemical Co. Ltd. (Zhuhai, China). All off the reagents were used as they were received if there was no special explanation.

### 2.2. Preparation of 3D Printing Ink

The 3D printing ink was prepared as two steps. Firstly, a 10% (*w*/*v*) alginate solution was prepared by adding sodium alginate into deionized water (DI water) at room temperature. Secondly, a certain amount of chitosan (as seen in Table 1) was added to the alginate solution and stirred sufficiently to ensure a good dispersion of chitosan in the alginate solution. To remove the air bubbles in the 3D printing ink, it was placed in vacuum drying oven at 30 °C for 30 min.

### 2.3. Preparation of 3D Printed Hydrogels

**Figure 1:**
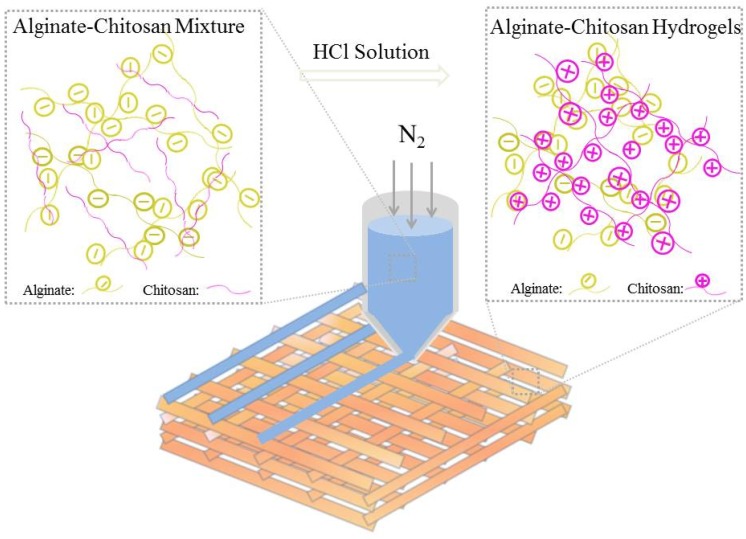
Schematic diagram of three-dimensional (3D) printing alginate–chitosan polyion complex hydrogels.

The 3D-BIOPLOTTER^TM^ (Envisionter Gmbh, Karlsruhe, Germany) with a resolution of 100 μm was used for the printing of all of the types of hydrogels. The 3D printing ink (Al1Ch0.2–Al1Ch1.2) was loaded into a dispensing unit consisting of a cartridge and a nozzle (400 μm), and was extruded out with applied nitrogen gas along the X–Y–Z target paths. The spacing between the two deposited fibers was set as 900 μm. After a layer of deposition was completed, the staying between the layers was 15 s. In the meantime, an HCl solution (0.5 mol/L) was sprayed on the deposited fibers with a single-use syringe, in order to protonate the NH_2_ on the chitosan. As the amino group was converted to ammonium, the 3D printed alginate–chitosan polyion complex hydrogels were prepared because the electrostatic interaction between the two oppositely charged polyelectrolytes (as shown in Figure 1). To verify the effects of the addition of chitosan on the morphology of, 3D printed alginate hydrogels were prepared in the same ways, except that the HCl solution was replaced with a CaCl_2_ solution (0.1 mol/L). If it was not specifically stated, the angle of layers of all of the hydrogels that were used for the characterization was 90°.

### 2.4. Rheological Test of 3D Printing Ink

Rheological properties of 3D printing ink with a different molar ratio of alginate to chitosan were measured on a rotary rheometer (AR-G2, TA, Waters, Newcastle, DE, USA), with parallel circular plates of a 40 mm diameter. Steady rate sweeps were conducted by varying the shear rates from 0.01 to 1000 s^−1^ at 30 °C, and the viscosity was measured at different shear rates.

### 2.5. Fourier-Transform Infrared Spectroscopy

A Fourier-transform infrared (FTIR) spectroscopy (Nexus Por Euro, Bruker, Karlsruhe, Germany) was used to verify the electrostatic interaction between alginate and chitosan. Using the FTIR spectra of alginate, chitosan, and 3D printed, the AlCh PIC hydrogel was obtained under the following conditions: the average of 32 scans between 400 cm^−1^ and 4000 cm^−1^ at a resolution of 4 cm^−1^.

### 2.6. Scanning Electron Microscope (SEM) Analysis

The structure and architecture of the 3D printed hydrogels was observed using an SEM (Quanta 200, FEI, Eindhoven, The Netherlands). The 3D printed hydrogels were prefrozen at −20 °C in a refrigerator and then freeze-dried in a freeze-drying machine (VIRTIS Genesis, Warminster, PA, USA). Before being mounted on aluminum stubs, they were treated with gold sputtering. The front and side morphology of the freeze-dried hydrogels were examined under SEM at an accelerated voltage of 10 KV, and the work distance (WD) was about 10 mm, which was adjusted according to the sample height.

### 2.7. Mechanical Properties

Compression tests of the rectangular block hydrogels with a size 10 mm × 10 mm × 3.2 mm were conducted on a universal material testing machine (Instron 5967, Instron, Norwood, MA, USA). The compression stress–strain curves were obtained when hydrogels were uniaxially compressed at a displacement rate of 1 mm/min to 90% strain. The compression strength and toughness were calculated through stress–strain curves. Compression stress with 90% strain represented the compression strength, while the toughness was equal to the area under the stress–strain curves. The compression modulus was calculated from the slope of the linear elastic region of the stress–strain curves, which was between 0% and 30% strain for all of the samples. In order to get the mean and standard deviation calculations, five parallel samples of each group were used for testing.

### 2.8. Swelling Ratio and Water Absorption of Hydrogels

For the swelling ratio and water absorption, rectangular block hydrogels of different internal structures, in size of 10 mm × 10 mm × 3.2 mm, were measured according to the reported methods by the authors of [15]. The 3D printed hydrogels were prefrozen at −20 °C in a refrigerator and then freeze-dried in a freeze-drying machine (VIRTIS Genesis, Warminster, PA, USA) to obtain freeze-dried hydrogels. The weight of the freeze-dried hydrogels was denoted as W_0_, and after that, they were immersed in phosphate buffered saline (PBS, pH = 7.4) at the 37 °C constant temperature using a shake table. At a fixed time, the PBS on the scaffold surface was wiped off, and the weight of hydrogels at the fixed time was denoted as W. In order to get the mean and standard deviation calculations, five parallel samples of each group were used for testing. The swelling ratio and water absorption of the hydrogels in equilibrium were calculated as the following:

$$\mathrm{Swelling}\;\mathrm{ratio}\;=\left(W\;-{\;W}_{0}\right)/W_{0}\times 100\%$$

$$\mathrm{Water}\;\mathrm{absorption}\;=\left(W\;-{\;W}_{0}\right)/W\;\times \;100\%$$

### 2.9. Degradation Properties of Hydrogels

Rectangular block hydrogels, with size 10 mm × 10 mm × 3.2 mm, were pre-weighted and immersed in PBS at 37 °C. At different time intervals, thee samples were taken out and rinsed with deionized water to remove the extra PBS (pH = 7.4) on the surface of the samples, and then, they were freeze dried and weighed again. The PBS solution was changed every 3 days. In order to get the mean and standard deviation calculations, five parallel samples of each group were used for testing. The degradation mass ratio was calculated as the following formula:

$$\mathrm{Degradation}\;\mathrm{mass}\;\mathrm{ratio}\;=\left(W_{1}\;-W_{2}\right)/W_{1}\times 100\%$$

where W_1_ is the initial weight of the hydrogel and W_2_ is final weight of the hydrogel.

### 2.10. Cell Viability Assay and Cell Morphology

Human adipose-derived stem cells (hASCs) were purchased from Cyagen Biosciences (Guangzhou, China) and were incubated to passage 5–10 in a culture medium (DMEM (dulbecco’s modified eagle medium) consisting of 10% FBS, (fetal bovine serum) 1% penicillin/streptomycin) at 37 °C in a constant temperature incubator. To seed the cells on the 3D printed AlCh PIC hydrogels, there were three steps. Firstly, the Al1Ch1.0 hydrogels, with size 10 mm × 10 mm × 2 mm, were freeze-dried with a freeze-dring machine and were placed in the 24-well plates to be sterilized by 15 KGy γ radiation. Secondly, the cell culture medium was added into the pores to immerse the dried hydrogels for 24 h in a super clean table. Thirdly, the cell culture medium was taken out, the material was air-dried in a super clean table for 30 min, and the cultured cells were dissociated with 0.25% trypsin-EDTA and centrifuged, and then, hASCs in 100 μL complete medium were seeded on each sample and incubated at 37 °C in a constant temperature incubator for 1 h. After this, 900 μL of the complete medium was added to each sample so as to give a cell density of 2 × 10^5^ cells per mL. The complete medium was changed every 2 days.

The cell viability of the constructs was examined by a Live–Dead viability Kit after the cells were cultured for 3 days. For the Live–Dead assay, firstly, the cell-laden hydrogels were washed with sterilized PBS 3 times; secondly, 100 μL of the live–dying stain was added to the pores; thirdly, 24-well plates were incubated at the 37 °C in a constant temperature incubator for 30 min; and finally, they were washed with sterilized PBS (pH = 7.4) 3 times. With the aid of a fluorescence inverted microscope, the live and dead cells that were distributed on the hydrogels were observed.

The morphology and adhesive properties of hASCs in the 3D printed hydrogels were observed by SEM (Quanta 200, FEI, Eindhoven, The Netherlands) after the cells were cultured for 3 days. The cells were fixed by glutaraldehyde (2.5%, *v*/*v*) overnight, after being washed with PBS, and they were dehydrated with an isocratic gradient ethanol solution. Then, they were dried in an air oven at 37 °C for 3 h and the morphology and adhesive properties of the hASCs were observed.

### 2.11. Proliferation of hASCs Cultured on Materials

The hASCs were incubated to passage 5–10 in a culture medium (DMEM consisting of 10% FBS, 1% penicillin/streptomycin) at 37 °C in a constant temperature incubator. Then, 2 × 10^5^/mL of hASCs in 100 μL were seeded on the sterilized Al1Ch1.0 hydrogels, with size 10 mm × 10 mm × 2 mm, and 1 h later, 900 μL of a culture medium was added to each sample, in order to give a cell density of 2 × 10^5^ cells per mL. Samples were taken out to place in another 24-well plate at fixed time, the proliferation of the hASCs were evaluated with a Cell Counting Kit-8 (CCK-8, Dojindo, Kumamoto, Japan), according to the manufacturer’s instruction. There was 1 mL of CCK-8 solution added to pores after hhe cells were incubated for 1 h at 37 °C in a constant temperature incubator. Finally, the optical density of the CCK-8 solution was measured at 450 nm using a microplate reader (Therm 3001, Ithaca, NY, USA). In order to get the mean and standard deviation calculations, five pores were used for testing.

### 2.12. Statistical Analysis

The data were expressed as mean ± standard deviation (SD). The statistical analysis was performed using the one way ANOVA (analysis of variance) test to determine significant differences. A *p*-value < 0.05 was considered as statistically significant.

## 3. Results and Discussion

### 3.1. Rheology of 3D Printing Ink

**Figure 2:**
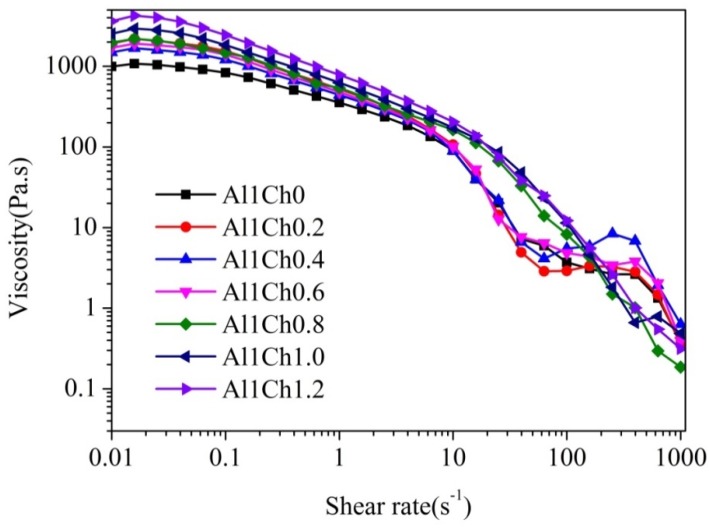
Rheology of 3D printing ink.

Rheology is the study of the flow of polymer solution under the application of shear stress. The study of the rheology properties of 3D printing ink is important for the 3D printed process. A high viscosity impedes both the droplet formation, resulting from surface tension force, and the collapse of deposited filaments. The shear thinning of the polymer solution is caused by shear-induced conformation changes in molecular chains from curly to stretched, which determines the continuous extrusion of 3D printing ink from the needle [2]. To study the effect of the content of chitosan on the viscosity of 3D printing ink, rheology tests of 3D printing ink were performed and the results are shown in Figure 2. When the concentration of the alginate solution increased to 10% (*w*/*v*), its further dissolution became more difficult, and the viscosity of the alginate solution could not be improved. However, a viscosity of 10% (*w*/*v*) in an alginate solution was insufficient for preparing hydrogels with complex structures, because of the collapse of deposited filaments. The chitosan was swelling but insoluble in alginate solution, in consequence, the viscosity of 3D printing ink prepared by adding chitosan powders into alginate solution can be improved. With the increased content of chitosan, Al1Ch1.2 had a viscosity of 3627 Pa·s, about four times that of Al1Ch0. As with the rheological test results of the sodium alginate solution, the viscosity of the 3D printing ink that was prepared by the addition of chitosan powders into an alginate solution decreased with the increased shear force. Therefore, an addition of chitosan into an alginate solution was effective for improving the viscosity of 3D printing ink, and had the potential to prepare hydrogels with complex structures.

### 3.2. Morphology of 3D Printed Hydrogels

**Figure 3:**
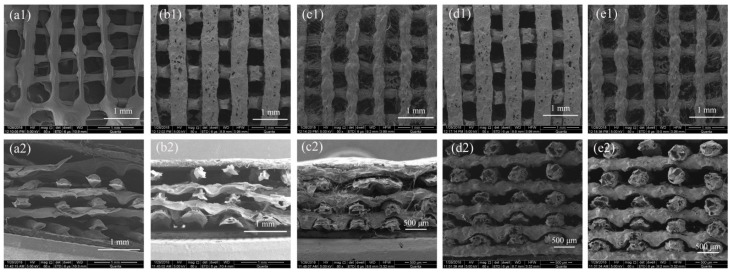
Morphological characterization of 3D printed hydrogels. Scanning electron microscope (SEM) micrographs of hydrogel prepared with 3D printing ink: (**a1**,**a2**) Al1Ch0; (**b1**,**b2**) Al1Ch0.2; (**c1**,**c2**) Al1Ch0.4; (**d1**,**d2**) Al1Ch0.6; and (**e1**,**e2**) Al1Ch0.8 (1 and 2 represent the front and side, respectively, of the 3D printed hydrogels).

The viscosity of 3D printing ink had an important influence on the structure of 3D printed of hydrogels. Because a high viscosity impeded the collapse of deposited filaments, the lateral pores of the 3D printed hydrogels were intact, and the height of hydrogels could also be larger than hydrogels that were prepared with low viscosity ink. To study the effect of 3D printing ink on the structure of 3D printed hydrogels, morphologies of freeze-dried hydrogels that were prepared with different 3D printing inks are shown in Figure 3. As a result of the relatively low viscosity of the 10% (*w*/*v*) alginate solution, the deposited filaments were easily collapsed, and the height of the 3D printed alginate hydrogel was difficult to be larger than 2 mm, which is seen in Figure 3(a2). As for the AlCh PIC hydrogels, as the chitosan swelled in the alginate solution, the increased viscosity was useful for preparing complex 3D printed hydrogels. The structure of hydrogels was improving with the increased content of chitosan in the 3D printing ink, as shown in Figure 3(b1–e2). The height of the 3D printed AlCh PIC hydrogels that were prepared with Al1Ch0.2–Al1Ch0.4 was difficult to be larger than 2 mm, moreover, the lateral pores were not obvious because of the collapse of the deposited filaments. However, the height of the 3D printed AlCh PIC hydrogels that were prepared with Al1Ch0.6–Al1Ch0.8 was getting larger than 3.5 mm. 3D printed hydrogels with a complex structure and high shape fidelity could be prepared when the molar ratio of chitosan to alginate was larger than 0.8.

**Figure 4:**
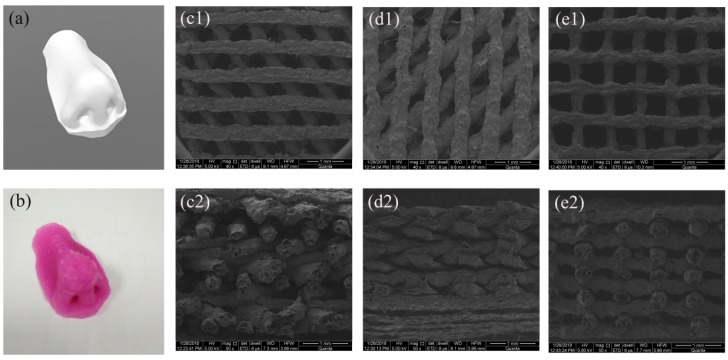
Morphological characterization of 3D printed hydrogels. Photographs of (**a**) 3D model of nose and (**b**) 3D printed nose prepared with 3D printing ink Al1Ch1.2. SEM micrographs of hydrogel with different angles between filaments, prepared with 3D printing ink Al1Ch1.0: (**c1**,**c2**) 45°; (**d1**,**d2**) 60°; (**e1**,**e2**) 90° (1 and 2 represent the front and side, respectively, of the scaffolds).

To verify that the 3D printing ink, which was prepared by adding a sufficient amount of chitosan into am alginate solution, was useful for printing hydrogels with complex structures, a nose model was built, after that, a 3D printed nose with a height of 20 mm was printed with Al1Ch1.2 printing ink (as seen in Figure 4b). From the photo of the 3D printed nose, it was consistent with the nose model that was used for 3D printed and had no collapse. From the 3D printed nose, we proved that the prepared 3D printing ink could be used to print organs with complex structures.

Under the condition of the same distance between adjacent filaments, the angle of filaments between layers led to the decrease of pore diameter, and thus, the hydrogels with different internal structures could be printed by changing the angle of the filaments between the layers. With the Al1Ch1.0 printing ink, we prepared the hydrogels with different internal structures by changing the angles between the layers, under the condition that the distance between the adjacent fibers was 0.9 mm (as shown in Figure 4(c1–e2)).

### 3.3. Fourier Transform Infrared Spectroscopy

**Figure 5:**
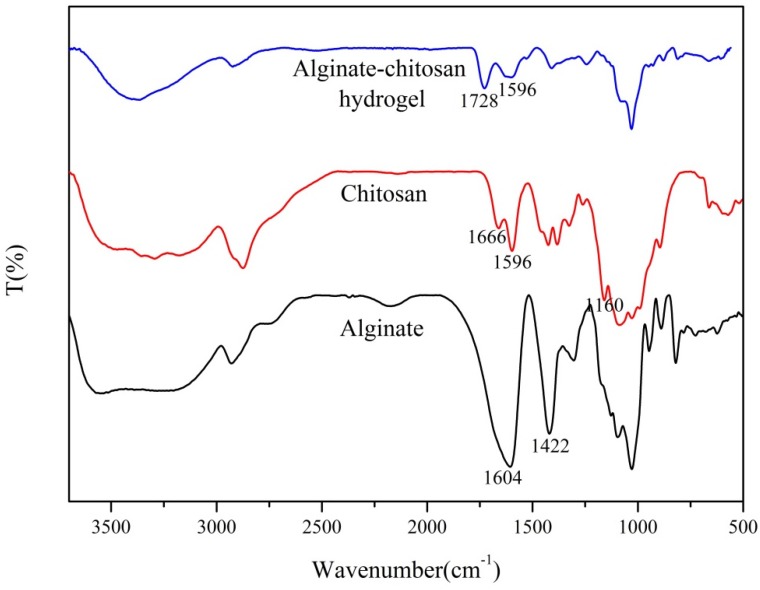
Fourier-transform infrared (FTIR) spectra of alginate and chitosan, and the 3D printed alginate–chitosan polyion complex hydrogels.

To verify that the 3D printed AlCh PIC hydrogels were formed by electrostatic interaction between two polyelectrolytes, the infrared absorption spectrum of alginate, chitosan, and the 3D printed AlCh PIC hydrogels (as shown in Figure 5) were analyzed. The characteristic peaks of the alginate were the peaks at 1604 cm^−1^ (C=O bond) and 1422 cm^−1^. The characteristic peaks of chitosan were the overlapped peaks at 1666 cm^−1^ (amide-Ι) and 1596 cm^−1^ (amide-II). The double amide peaks in the spectrum of chitosan corresponded to the partial N-deacetylation of the chitin [16]. In the spectrum of the AlCh PIC hydrogels, the amide-Ι peaks shifted from 1666 cm^−1^ to 1728 cm^−1^, while the amide-peaks were significantly intensified. On the other hand, the amino peak at 1160 cm^−1^ and the carboxyl peak at 1604 cm^−1^ disappeared in the spectrum of the AlCh PIC hydrogel, which all suggested the electrostatic interaction between alginate and chitosan. Our results were similar to other authors’ work [17,18], who had prepared scaffolds by an electrostatic interaction between alginate and chitosan.

### 3.4. Mechanical Properties of 3D Printed AlCh PIC Hydrogels

**Figure 6:**
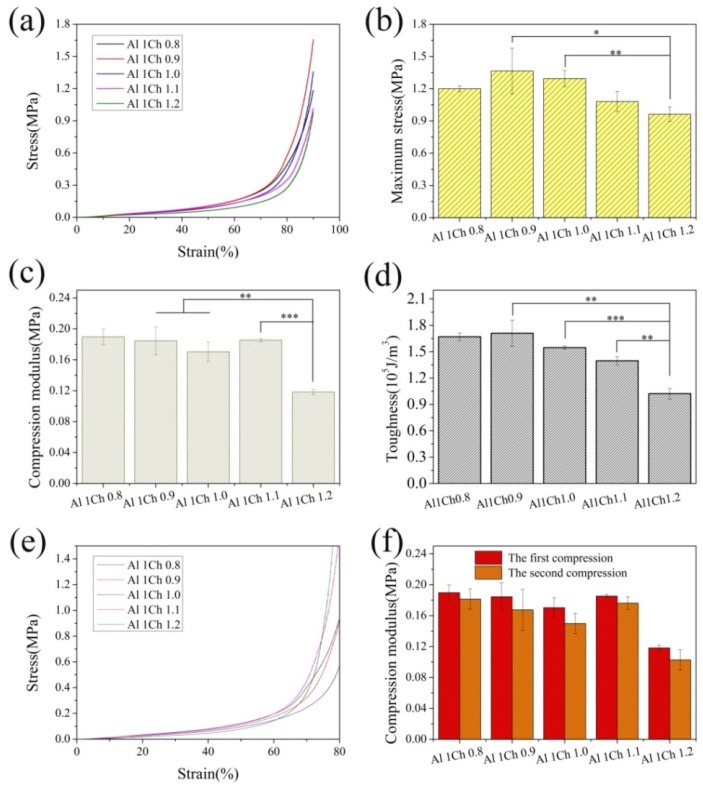
Compressive properties of 3D printed alginate–chitosan polyion complex (AlCh PIC) hydrogels prepared with different molar ratio of alginate to chitosan. The (**a**) compression stress–strain curves; (**b**) maximum stress at 90% strain; (**c**) compression modulus; (**d**) toughness; (**e**) compression stress–strain curves; and (**f**) compression modulus of the two compressions.

The mixing of bulk solutions of polycation and polyanion usually led to inhomogeneous precipitation, where strong polyion complexes (PIC) were formed at the interface of the two solutions, which quenched the further reaction. Because of the limited reactive ion pairs that were involved in the reaction, the crosslinking density of the biomaterial internal network was low, and so the mechanical properties of the biomaterial were made by adding the chitosan powders into an alginate solution, and then the alginate–chitosan mixture was treated with an HCl solution. The changes in pH were used to induce a poly-complexation of the two polyelectrolytes. A molecule of HCl was a small molecule that diffused easily, thus, the further reaction between the two oppositely charged polyelectrolytes would not be quenched. With the increase of the reactive ion pairs, the mechanical strength of the 3D printed AlCh PIC hydrogels could be improved. At a 10% (*w*/*v*) concentration of the alginate solution, the content of chitosan had an effect on reactive ion pairs, and thus affected the mechanical properties of the 3D printed AlCh PIC hydrogels. To explore the effect of the chitosan content on the mechanical properties of 3D printedg AlCh PIC hydrogels, compression tests were conducted towards the 3D printed AlCh PIC hydrogels with different molar ratios of chitosan to alginate, and the results are shown in Figure 6. There was no significant difference in the mechanical properties between the hydrogels whose ratio of chitosan to alginate was 0.9–1.1, but the mechanical properties of Al1Ch1.2 became poor and had a significant difference with the hydrogels that were prepared with the Al1Ch0.9–Al1Ch1.1 ink. When the molar ratio of chitosan to alginate was 0.9–1.1, the number of positive and negative charges that were involved in the reaction was basically the same. When the content of the chitosan increased further, the electrostatic repulsion between the excessive positive charges would affect the electrostatic interaction between opposite charges, thus leading to the decrease of the mechanical properties of the 3D printed hydrogels. For 3D printed AlCh PIC hydrogels, the maximum compression stress at 90% strain, compression modulus, and toughness could reach 1.4 MPa, 0.2 MPa, and 1.8 × 10^5^ J/m^3^, respectively.

When the compression tests were completed at 90% strain, the hydrogels were not broken. After being soaked in deionized water for 1 h, the hydrogels were taken out for the second compression. The two compression stress–strain curves and modulus are shown in Figure 6e,f. There was no significant difference between the two compression modulus, which indicated that 3D printed AlCh PIC hydrogels had a good fatigue resistance.

The compression strength of 3D printed hydrogels (Al1Ch0.9) that were prepared by crosslinking 10% alginate and 7% chitosan, could achieve 1.5 MPa, which was about the same as the bulk hydrogel that were prepared by photo crosslinking 20% PNAGA (*N*-acryloyl glycinamide), and the much larger than bulk and 3D printed hydrogels that were prepared by adding 7% clay into a 10% PNAGA photo crosslinking network [19]. The compression modulus of the hydrated scaffolds that were prepared by alginate and chitosan were 83.1 ± 14.6 KPa [20], which was far lower than the 3D printed hydrogels that were prepared by alginate and chitosan.

### 3.5. Swelling Ratio and Water Absorption of Hydrogels

**Figure 7:**
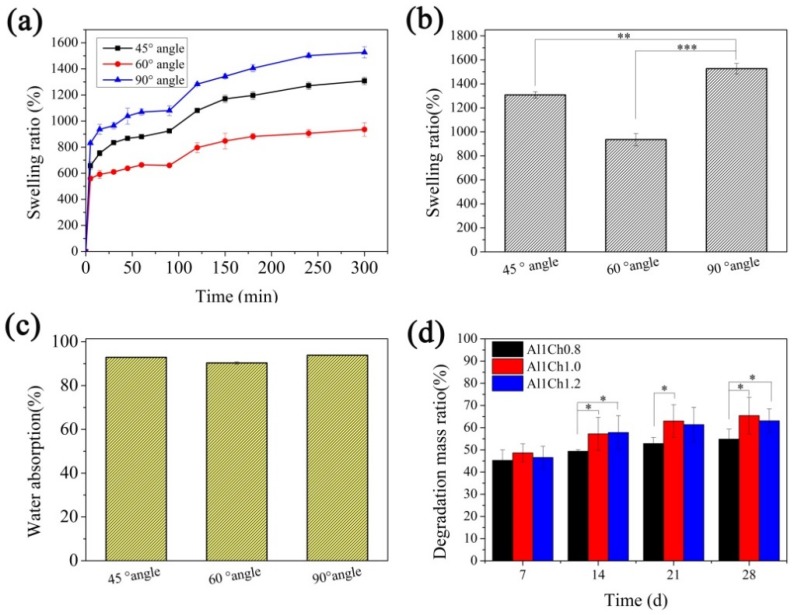
Physiochemical properties of 3D printed AlCh PIC hydrogels. (**a**) swelling process; (**b**) swelling ratio; (**c**) water absorption; (**d**) degradation mass ratio of hydrogels.

As tissue engineering scaffolds, the swelling of the hydrogels helped to provide nutrients and promote the metabolism during the cell culture. The swelling process was greatly influenced by the internal pore structure of the 3D printed hydrogels. We prepared Al1Ch1.0 hydrogels with different internal structures, by changing the angle of layers, and then, the swelling ratio of the hydrogels with different internal structures at each time point were tested (as seen in Figure 7a). The swelling ratio and water absorption rate in the equilibrium state of swelling were concluded and, as shown in Figure 7b,c, all of the hydrogels had a good swell behavior. The swelling ratio of the 3D printed hydrogels with different internal structures were different, because the swelling ratio was affected by the porosity of the hydrogel, while the porosity of the 3D printed hydrogels was affected by the angle of the layers. When the angle of the layers was 90°, the porosity of the 3D printed AlCh PIC hydrogels was maximum as well as for the swelling ratio, and they had a significant difference with swelling ratio of the hydrogels whose angle of layers were 45° and 60°. The maximum amount of absorbed water was 15 times the dry weight of the hydrogels. There was no significant difference between the water absorption rate of the hydrogels with a different internal structure (as shown in Figure 7c), as water accounted for most of the weight of all of the hydrogels in the equilibrium state of swelling.

### 3.6. Degradation Properties of Hydrogels

A suitable degradation rate was important for the tissue engineering scaffolds, when the degradation was too fast, the scaffolds lost their role of supporting for the cells. In contrast, when the degradation rate ws too slow, it would have impeded the emergence of the neo tissue. When alginate was crosslinked through Ca^2+^, calcium ions were easily replaced by monovalent cation, and the hydrogels in vivo could not exist stably. However, when the alginate was crosslinked with chitosan, because of the entanglement between the molecular chains of alginate and chitosan, the crosslink networks were more stable, so the AlCh PIC could exist for a long time. Furthermore, through adjusting the ratio of the alginate to chitosan, we had a control over the degradation rate of the hydrogels. Figure 7d shows the degradation process of the 3D printed AlCh PIC hydrogels. As time went on, the dry mass of the hydrogels decreased, and the degradation mass ratio of the different hydrogels at 28 days was 50–70%. The content of the chitosan had an effect on the degradation properties because of the different crosslink density of the hydrogels. There were significant differences between the degradation mass ratios of the different hydrogels. On the other hand, we measured the mechanical modulus change of the bulk alginate hydrogel that was crosslinked with Ca^2+^ and the bulk AlCh PIC hydrogels, and it could be seen obviously that the AlCh PIC was more stable than the alginate hydrogels that were crosslinked with Ca^2+^ (seen in Figure S1). On the 12th day, the alginate hydrogel that was crosslinked with Ca^2+^ degraded absolutely, while the AlCh PIC hydrogels were still tough, the compression stress of the AlCh PIC hydrogels was 0.3–0.4 MPa by the 16th day.

### 3.7. Biocompatibility of Hydrogels

**Figure 8:**
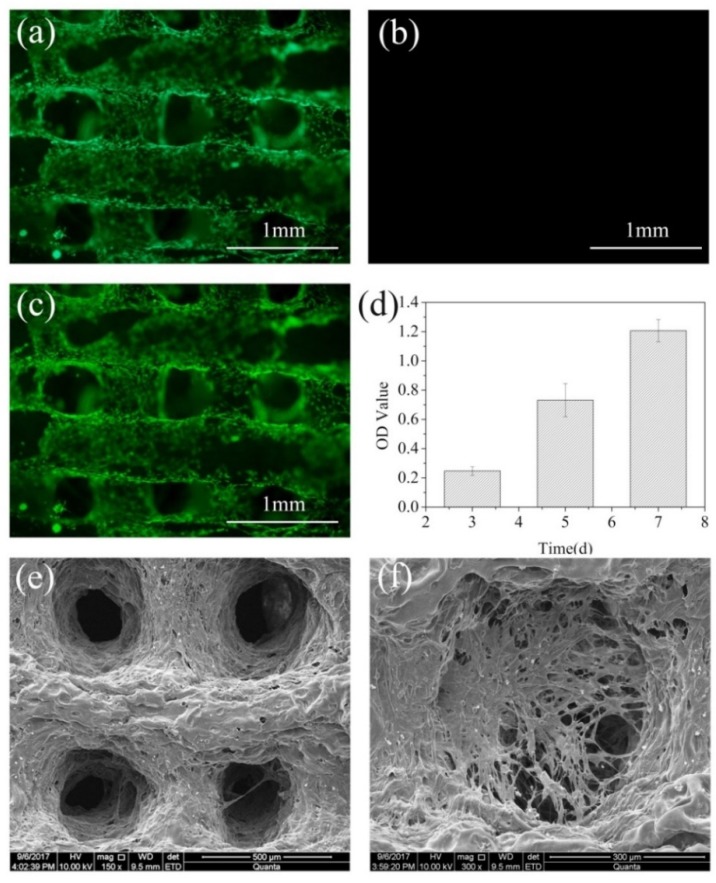
Biocompatibility of 3D printed AlCh PIC hydrogels. Inverted fluorescence microscope of (**a**) live; (**b**) dead; (**c**) merged cells at the 3rd day. (**d**) Proliferation of hASCs cultured on 3D printed AlCh PIC hydrogels. (**e**,**f**) SEM micrographs of 3D printed AlCh PIC hydrogels after cultured with hASCs for 3 days.

Stem cells are pluripotent cells, and human adipose-derived stem cells (hASCs) were obtained from adipose tissue. The use of hASCs was feasible, as the adipose tissue was easily available. Furthermore, the hASCs expressed the biochemical profile of adipocytes, chondrocytes, and osteoblasts under the appropriate conditions [21]. The hASCs were used to test the biocompatibility of the hydrogels. Figure 8a–c shows the live–dead cells that were distributed on the hydrogels on the third day. It could be seen that the live cells spread uniformly on the hydrogels, while little or no dead cells existed, which showed the 3D printed AlCh PIC hydrogels’ biocompatibility. On the other hand, the hASCs that were distributed on the materials proliferated with time, which was shown in Figure 8d. Figure 8e,f shows the morphology of the cells on the hydrogels on the third day, where the cells adhered to the 3D printed AlCh PIC hydrogels, and expanded along the direction of the deposited filaments.

## 4. Conclusions

In this study, we prepared 3D printed hydrogels by changing the pH so as to induce the poly-complexation of the alginate and chitosan after the 3D printing ink was extruded out. The addition of chitosan into the alginate solution ws useful for improving the viscosity of 3D printing ink. Moreover, the molecule of HCl was a small molecule that diffused easily, and the gelation process, through the change of pH to induce poly-complexation between the alginate and chitosan, was fast. Thus, the deposited filaments were not easily collapsed or fused, and complex constructs could be prepared through this method. The physicochemical properties of the obtained hydrogels could be controlled by controlling the content of chitosan. By printing the nose with the alginate–chitosan mixture, we proved that the prepared 3D printing ink could be used to print tissues or organs with complex structures. It was possible to fabricate other tissues or organs with this alginate–chitosan mixture.

Finally, hASCs were pluripotent cells. We verified that the hASCs that were seeded on the 3D printed AlCh PIC hydrogels adhered and proliferated, so the methods in this work could prepare hydrogels with a complex structure, to provide cells with appropriate growing environment. A further induced differentiation of hASCs in vitro could obtain neo tissue to repair or even replicate damaged tissues, thus the 3D printed AlCh PIC hydrogels had potential to be used for tissue engineering.

## Figures and Tables

**Table 1:** Three-dimensional (3D) printing ink made from different molar ratio of alginate to chitosan.

3D Printing Ink	Alginate (g)	Chitosan (g)	Water (mL)	Alginate:Chitosan (mol:mol)
Al1Ch0	2	0	20	1:0
Al1Ch0.2	2	0.333	20	1:0.2
Al1Ch0.4	2	0.665	20	1:0.4
Al1Ch0.6	2	0.998	20	1:0.6
Al1Ch0.8	2	1.331	20	1:0.8
Al1Ch0.9	2	1.497	20	1:0.9
Al1Ch1.0	2	1.663	20	1:1.0
Al1Ch1.1	2	1.830	20	1:1.1
Al1Ch1.2	2	1.996	20	1:1.2

## Supplementary Materials

The following are available online at http://www.mdpi.com/2073-4360/10/6/664/s1, Figure S1: Compression modulus of bulk hydrogels during the degradation process.
