# Supplementary material for: Preparation and Properties of 3D Printed Alginate–Chitosan Polyion Complex Hydrogels for Tissue Engineering
Source: Polymers (Basel). 2018 Jun 14;10(6):664. doi: 10.3390/polym10060664 (PMC6404366; doi:10.3390/polym10060664)
Supplement: Supplementary file 1 [file polymers-10-00664-s001.zip › Supplementary Files/Supplementary Files.docx]

**Supplementary materials:**


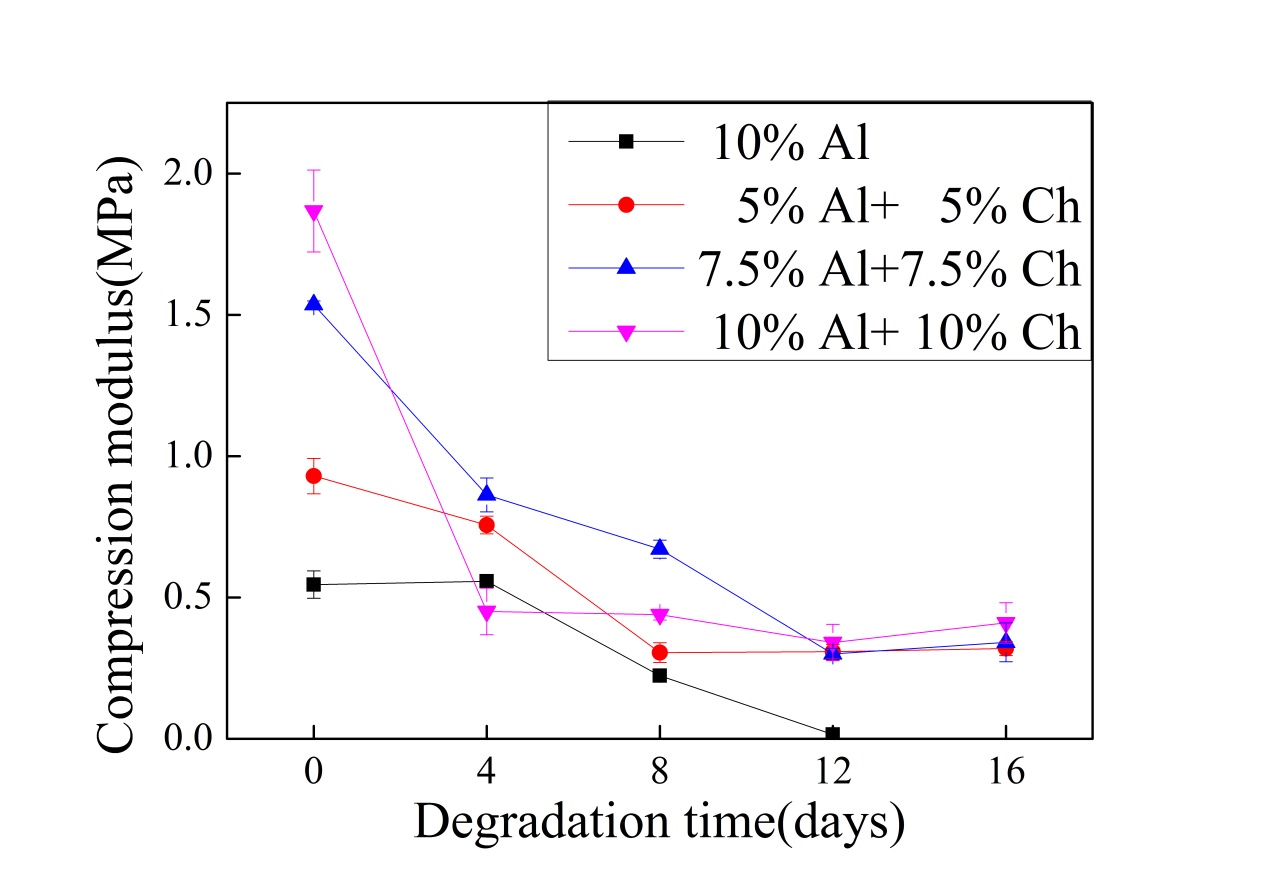


Figure S1: Compression modulus of bulk hydrogels during the degradation process.
