# Supplementary figures and images for: Preparation and Properties of 3D Printed Alginate–Chitosan Polyion Complex Hydrogels for Tissue Engineering
Source: Polymers (Basel). 2018 Jun 14;10(6):664. doi: 10.3390/polym10060664 (PMC6404366; doi:10.3390/polym10060664)

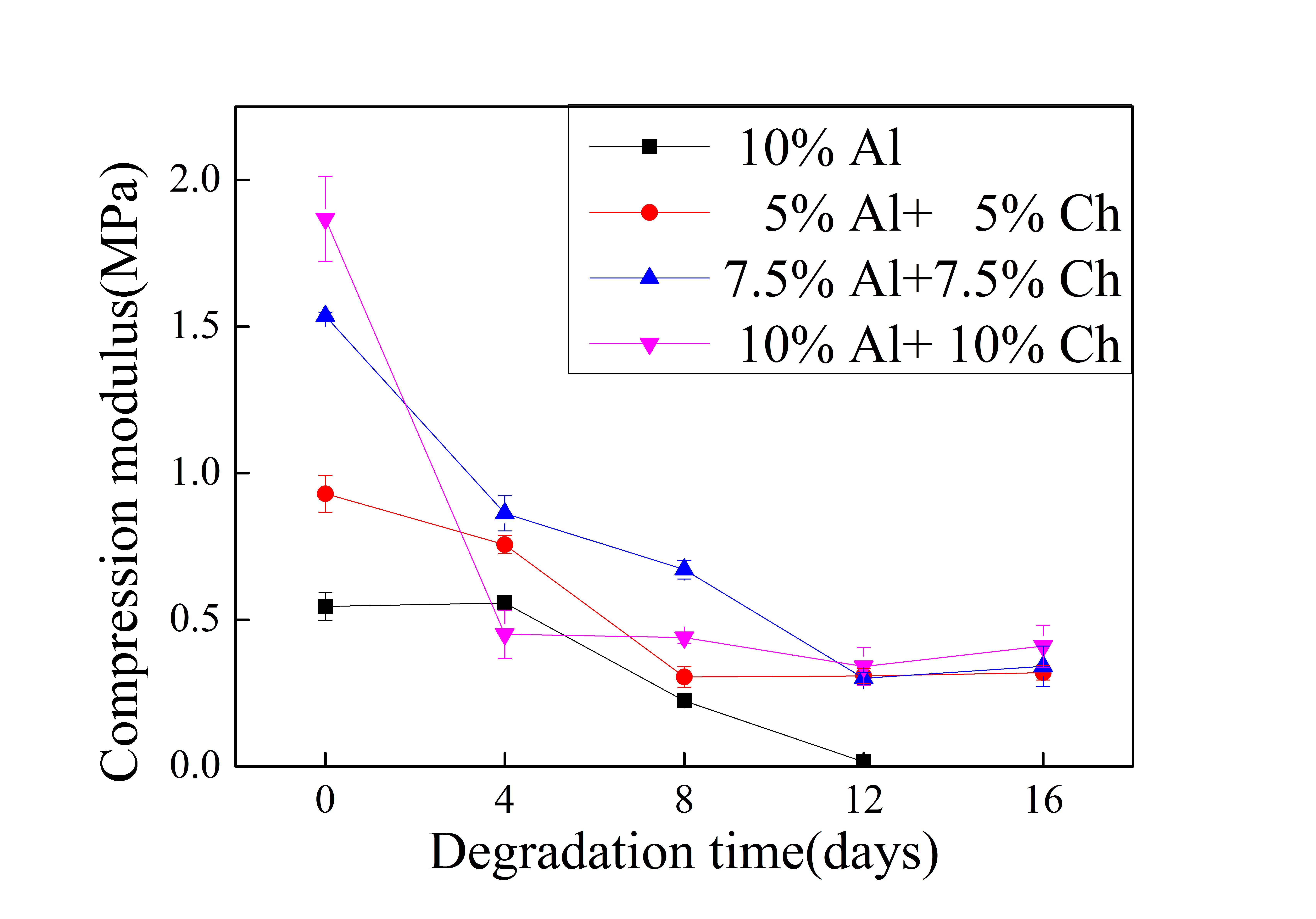

Supplement: Supplementary file 1 [file polymers-10-00664-s001.zip › Supplementary Files/Fig S1.tif]
